# Method Specific Calibration Corrects for DNA Extraction Method Effects on Relative Telomere Length Measurements by Quantitative PCR

**DOI:** 10.1371/journal.pone.0164046

**Published:** 2016-10-10

**Authors:** Luise A. Seeker, Rebecca Holland, Sarah Underwood, Jennifer Fairlie, Androniki Psifidi, Joanna J. Ilska, Ainsley Bagnall, Bruce Whitelaw, Mike Coffey, Georgios Banos, Daniel H. Nussey

**Affiliations:** 1 Animal & Veterinary Sciences, SRUC, Roslin Institute Building, Easter Bush, Midlothian EH25 9RG, United Kingdom; 2 The Roslin Institute and Royal (Dick) School of Veterinary Studies, University of Edinburgh, Easter Bush, Midlothian, United Kingdom; 3 Institute of Evolutionary Biology, School of Biological Sciences, University of Edinburgh, Edinburgh, Midlothian, United Kingdom; 4 SRUC Crichton Royal Farm, Glencaple Road, Dumfries, United Kingdom; Centre National de la Recherche Scientifique, FRANCE

## Abstract

Telomere length (TL) is increasingly being used as a biomarker in epidemiological, biomedical and ecological studies. A wide range of DNA extraction techniques have been used in telomere experiments and recent quantitative PCR (qPCR) based studies suggest that the choice of DNA extraction method may influence average relative TL (RTL) measurements. Such extraction method effects may limit the use of historically collected DNA samples extracted with different methods. However, if extraction method effects are systematic an extraction method specific (MS) calibrator might be able to correct for them, because systematic effects would influence the calibrator sample in the same way as all other samples. In the present study we tested whether leukocyte RTL in blood samples from Holstein Friesian cattle and Soay sheep measured by qPCR was influenced by DNA extraction method and whether MS calibration could account for any observed differences. We compared two silica membrane-based DNA extraction kits and a salting out method. All extraction methods were optimized to yield enough high quality DNA for TL measurement. In both species we found that silica membrane-based DNA extraction methods produced shorter RTL measurements than the non-membrane-based method when calibrated against an identical calibrator. However, these differences were not statistically detectable when a MS calibrator was used to calculate RTL. This approach produced RTL measurements that were highly correlated across extraction methods (r > 0.76) and had coefficients of variation lower than 10% across plates of identical samples extracted by different methods. Our results are consistent with previous findings that popular membrane-based DNA extraction methods may lead to shorter RTL measurements than non-membrane-based methods. However, we also demonstrate that these differences can be accounted for by using an extraction method-specific calibrator, offering researchers a simple means of accounting for differences in RTL measurements from samples extracted by different DNA extraction methods within a study.

## Introduction

Telomere shortening has recently been identified as one of nine ‘hallmarks of aging’ [1] and blood cell telomere length (TL) is an increasingly widely measured biomarker in human epidemiology and vertebrate ecology [2–4]. Many methods are available to measure TL, each with their own strengths and drawbacks [5,6]. Quantitative PCR (qPCR)-based methods have become increasingly popular in recent years, presumably due to their being faster, cheaper and requiring less DNA than most other methods [5,6]. However, the qPCR method has drawbacks, notably a lower repeatability compared to terminal restriction fragment (TRF) southern blot [7,8] and the relative units of measurement, which makes comparison across studies and species extremely challenging [5,7] if not impossible. Furthermore, there is mounting recent evidence that relative TL (RTL) measurements by qPCR may be influenced by methods of sample acquisition and storage [9] and DNA extraction methods [10–14]. Understanding how such methodological variation may influence RTL measurements by qPCR both within and among laboratories is essential for evaluating and comparing results of telomere studies.

A central requirement of all methods of TL measurement is the extraction of a suitable quantity of high quality DNA. A considerable number of DNA extraction methods have been employed to date by researchers studying TL [10]. In general two different types of DNA extraction methods can be distinguished: One uses a solid phase such as silica membranes or magnetic beads. DNA binds to the solid phase, is washed and then eluted. The other type is based on the transition of DNA between different solvents. Those methods (for example salting out or phenol-chloroform extractions) do not require a solid phase. The question that arises from the literature is whether solid phases act as physical barriers that shear DNA and therefore cause shorter TL measurements. Two recent studies using human blood samples with the qPCR method suggested that silica membrane-based DNA extraction methods yield shorter RTL measurements than other methods [10,11]. Two further studies have reported differences in mean TL from DNA extracted using a range of different methods, although these differences were not specifically linked to the use of silica membranes [12,13]. Recently, another study found that RTL from samples extracted by a magnetic bead method was shorter when compared to salting out and phenol chloroform [14]. Although it is obviously desirable to keep methodology as consistent as possible, potentially valuable and informative archived DNA samples may be available to researchers interested in telomere dynamics which may not have been extracted by the same technique. In such cases, understanding and potentially accounting for the effects of extraction method on TL measurement is essential [15]. Furthermore, a better understanding of such methodological effects could help ensure appropriate aspects of DNA preparation methodology are accounted for in meta-analyses of TL studies [10].

The qPCR method measures RTL as the total amount of telomeric sequence relative to the amount of a non-variable copy reference gene sequence within the same DNA sample [16]. Standard methods for calculating RTL require a calibrator sample (also called “reference sample” [16] or “golden sample” [6]), which is an identical DNA sample included on every qPCR plate for both telomere and reference gene reactions. Sample RTL is expressed relative to the calibrator to account for random measurement error and resulting plate-to-plate variation. A wide range of samples have been used as calibrators: DNA from a chosen individual, pooled DNA from several individuals [16] or commercially available DNA [14]. Previous studies examining effects of DNA extraction method on RTL appear to have used a single calibrator, extracted by one identical method [10–14]. They observed extraction method dependent differences in RTLs that in some studies appear to be not random but systematic [10,11,14]. In principle, it should be possible to account for such systematic extraction method effects by taking the same calibrator sample and extracting DNA from it using different methods to match the methods used on the samples in the study. With this approach, the calibrator should be influenced in the same direction and to a similar degree by the extraction method. Using such a DNA extraction method specific calibrator in RTL calculations, could therefore adjust for any effect of extraction method on the samples’ telomere length. The effectiveness of this approach has yet to be tested.

The objective of the present study was to assess the effect of two different DNA extraction methods, and the use of different calibrators on RTL measurements. We compared RTL measurements of blood samples that were collected from a Holstein Friesian cattle population after extracting DNA using two silica membrane-based DNA extraction protocols and a salting out (non-membrane-based) method. To validate our results with samples from a different species we compared one of the two silica membrane-based methods with the salting out method using buffy coat samples from wild Soay sheep. We found high repeatability of RTL measurements, regardless of DNA extraction method, and no difference in mean RTL among extraction methods when a DNA extraction method specific (MS) calibrator was used.

## Materials and Methods

### Study systems & sampling

Whole blood samples were collected from Holstein Friesian cattle during 2009–2013 at the Crichton Royal Farm (Dumfries, Scotland) as part of a long-term genetics study for which blood samples have been archived for many years [17]. Samples were taken by venepuncture using EDTA as anticoagulant and were stored at -30°C until DNA extraction. We selected 72 samples from animals among which both sexes and a range of ages were represented (45 females aged 0–9 years and 27 male new-born calves).

Additionally, we used blood samples collected from a wild population of Soay sheep on the St Kilda archipelago in the Outer Hebrides (Scotland), which have been subject to individual-based monitoring and regular sampling since 1985 [18]. Blood samples were taken by venepuncture in August 2013, using heparin as an anticoagulant. Buffy coat fractions were prepared as follows: whole blood samples were centrifuged at 3,000 rpm for 10 minutes. The plasma layer was removed and remaining cells were washed by adding 0.9% NaCl solution. After centrifugation for 10 minutes at 3,000 rpm the intermediate buffy coat layer was collected, transferred to a 1.5 ml Eppendorf tube and stored at -20°C until further use. We selected samples from 48 different females aged 4–13 years for DNA extraction.

### Ethics statement

Blood sampling from Holstein Friesian cattle and Soay sheep was approved by the Animal Experiments Committee (UK Home Office Project License Numbers: PPL 60/4278 and 60/3547, respectively).

### DNA extraction

DNA from each cattle sample was extracted using the QIAGEN Gentra Puregene kit (PG) based on a non-membrane salting out method and two silica membrane-based protocols of the QIAGEN DNeasy Blood & Tissue kit: spin column (SC) and the 96-well plate (SP). DNA from each sheep sample was extracted using the PG and SC protocols.

According to the PG protocol, DNA is first isolated by removing red blood cells and lysing white blood cells. RNA and proteins are removed by enzyme digestion and salt precipitation, respectively. DNA is recovered by alcohol precipitation and dissolved in DNA hydration solution. The SC and SP protocols rely on a silica-based extraction method during which cells are lysed and transferred onto silica membranes to which DNA binds specifically during a centrifugation step. DNA is washed and finally eluted using a DNA hydration buffer. When possible, we performed different DNA extraction methods simultaneously on each sample. We followed the manufacturer’s protocol with certain alterations to improve yield and quality of DNA samples. The most important alternation was that the silica protocols were started with a red blood cell lysis step that allowed us after centrifugation to transfer only the white blood cell pellet dissolved in PBS onto the silica membranes. This step removed impurities in the beginning of the protocol and improved purity measurements greatly. SC samples were also prepared in duplicates that were run through the same silica membrane to improve DNA yield. All alternations are detailed in S1 File. Fifteen cattle samples extracted by PG had to be re-purified following appendix C of the manufacturer’s manual.

### Quality control of DNA extracts

We employed a strict quality control (QC) strategy during DNA extraction and qPCR to ensure that samples extracted by different methods were of similar quality, purity and integrity. Our aim was to minimize the risk of differences between DNA extraction measurements being due to sample quality rather than differences of methods themselves. Samples failing QC were excluded from our final analyses (Table 1).

**Table 1:** Number of samples after each quality control step by species and method of DNA extraction.

Quality control step	DNA extraction method				
	Cattle			Sheep	
	PG	SC	SP	PG	SC
**1.** Starting samples	72	72	72	47	47
**2.** DNA yield >20ng/ul on Nanodrop	66	71	66	47	47
**3.** Protein contamination (260:280 ratio > 1.7)	66	71	66	47	41
**4.** Salt contamination (260:230 ratio > 1.8)	61	71	66*	47	39
**5.** DNA yield >20ng/ul on Qubit/FLUOstar	61	71	62	47	39
**6.** DNA integrity score <3	61	69	56	47	36
7. Sample selection (samples passing all tests for all methods)	56	56	51	36	36
8. Number of RTL measurements (sample number x qPCR plates)	224	224	196**	144	144
9. qPCR efficiencies for each triplicate within 5% of mean plate efficiency	224	223	196	144	144
10. Triplicate sample Cq values had CV < 5%	223	221	196	142	144

PG = Gentra Puregene Kit; SC = Spin Column protocol (DNeasy Blood & Tissue Kit); SP = Spin Plate protocol (DNeasy Blood & Tissue Kit)

* This step did not apply to SP.

**Four samples were run on two qPCR plates only, because they did not yield enough DNA for more measurements.

We tested DNA yield and purity using a NanoDrop ND-1000 spectrophotometer (Thermo Scientific) with the software NanoDrop 2000. Samples with DNA concentrations less than 20 ng/μl were excluded from further investigation (Table 1). The average ratio of absorbance at 260 nm over 280 nm (OD 260/280) over two measurements was used to check for protein contamination and the average ratio at 260nm over 230nm (OD 260/230) was used to check for salt contamination. Both proteins and some salts can act as qPCR inhibitors [19]. Extracts with OD 260/280 < 1.7 or OD 260/230 < 1.8 were excluded from further analyses for PG and SC methods. For SP, OD 260/230 readings were variable probably due to samples with low yields approaching the limit for accurate contaminant detection. We therefore decided not to exclude SP samples based on OD 260/230, although we applied the same OD 260/280 QC threshold as for the other methods. Note that results obtained from SP extracted samples behaved very similarly to the SC samples, despite the variable OD 260/230 ratios (see Results).

**Fig 1:**
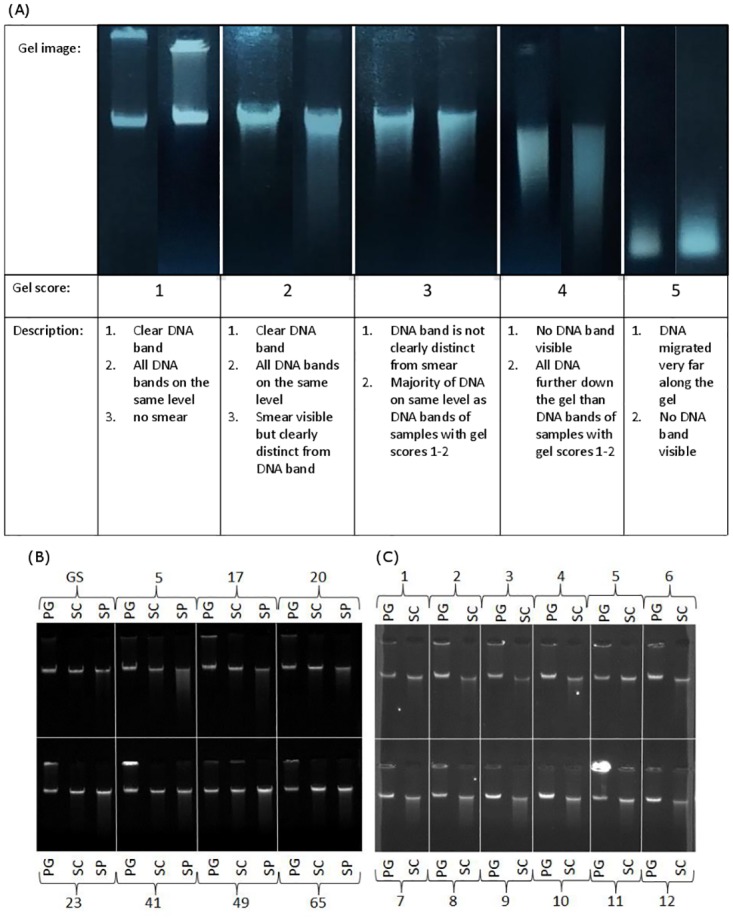
DNA integrity gels. (A) Illustrative DNA Integrity gels with gel scores. Example integrity gels for (B) Holstein Friesian cattle and (C) Soay sheep. Individual samples (represented by numbers in image) that were extracted with different DNA extraction protocols. (PG: Gentra Puregene kit, SC: DNeasy spin columns, SP: DNeasy 96 well plate; GS: calibrator DNA (“golden sample”).

To assess DNA concentrations more accurately all PG and SC extracts were subsequently measured on a Qubit^®^ 2.0 (Invitrogen) using a Qubit^®^ dsDNA BR Assay kit (Invitrogen) according to the manufacturer’s manual. SP extracts were measured on a FLUOstar Galaxy microplate reader (BMG LABTECH) using a Quant-iT^™^ dsDNA Assay Kit (Invitrogen) following the manufacturer’s instructions. Both procedures are based on the detection of a fluorophore that becomes fluorescent when bound to double stranded DNA. Measurements are evaluated in relation to standards with known DNA concentrations. Because the signal is specific for double stranded DNA (dsDNA) fluorescence spectroscopy measurements are more accurate for DNA yield than NanoDrop measurements. Samples with average concentrations lower than 20 ng/μl calculated over two measurements on either fluorometer were excluded from further investigation. DNA integrity was assessed visually by running 200ng on a 0.5% agarose gel with ethidium bromide at a final concentration of 0.8 μg/ml. Gels were run at 100 mV and 200 mA for 45 minutes and then visualised with an AlphaImager TM 2200. Gels were visually scored for integrity on a scale of 1 to 5 (Fig 1A) and extracts with a score greater than 2 were removed from further analyses. DNA stock solutions were prepared by diluting extracts to a concentration of 10 ng/μl based on fluorescence measurements. PG extracts were diluted in DNA hydration solution (QIAGEN), and SC and SP extracts were diluted in buffer AE (QIAGEN).

### Telomere length measurement

Leukocyte RTL was measured by qPCR [16] as the amount of telomeric DNA in a sample relative to the amount of a non-variable copy number reference gene. In order to identify the most appropriate reference gene we conducted preliminary analyses considering a variety of candidate reference gene primer pairs. The most consistent amplification profile and cleanest melting curve was obtained in both species using Primerdesign primers targeting the beta-2-microglobulin (*B2M*) gene (accession number: NM_001009284), which we selected as our reference gene. The selection of our reference gene was based on comparison of a panel of 12 candidate genes for sheep and 6 for cattle, supplied as part of the Primerdesign GeNorm kit (following Fairlie et al. 2016). B2M showed completely stable qPCR results indicative of non-variable copy number, and is well conserved and located on chromosome 10 of the bovine genome and chromosome 7 of the ovine genome [20,21]. For the telomere amplification, tel 1b (CGG TTT GTT TGG GTT TGG GTT TGG GTT TGG GTT TGG GTT) and tel 2b (GGC TTG CCT TAC CCT TAC CCT TAC CCT TAC CCT TAC CCT) primers were used [22]. Telomere primers were manufactured and purified with high performance liquid chromatography by Integrated DNA Technologies (IDT, Glasgow, UK).

The use of identical primers allowed us to use identical reaction conditions for both cattle and sheep qPCRs. We ran samples extracted by different methods and species on separate 384-well plates. Reactions for telomere and *B2M* primers were run in separate wells (monoplex qPCR) but on the same qPCR plate. Each qPCR plate was repeated four times over two days. Our calibrator sample came from a large volume of blood obtained from an individual cow or sheep, respectively. We extracted large quantities of DNA from each calibrator sample using different methods to match those applied to our experimental samples: PG, SC and SP for cattle, PG and SC for sheep. In the cattle experiment, each qPCR plate included three calibrator samples, one for each of the extraction methods used (i.e., calibrator samples extracted with PG, SC and SP methods). In the sheep experiment, we only included the MS calibrator on each plate (i.e. PG-extracted calibrator on plates of PG-extracted samples and SC-extracted calibrator on plates of SC-extracted samples).

Samples and calibrators were loaded at a dilution of 1 ng/μl onto a 96 well plate (sample plate) that also contained a four step 1:4 serial dilution of calibrator DNA starting with 10 ng/μl as standard and nuclease free water as non-template control. A Freedom EVO 2150 robot (by TECAN) was used to transfer all samples, standards, calibrators and negative controls in triplicate onto a 384 well qPCR plate. The robot mixed 1 μl of the contents of the sample plate with 9 μl of master mix in each qPCR plate well. The master mix for both reactions contained 5 μl of LightCycler 480 SYBR Green I Master (Roche) per well. Telomere primers were used at a concentration of 900 nmol, *B2M* primers were used at 300 nmol. Nuclease-free water was added to the master mix to have a final volume of 10 μl per well.

The qPCR was performed on a LightCycler 480 (Roche) using the following protocol: Enzyme activation: 15 min at 95°C; then 50 cycles of: 15 s at 95°C (denaturation), 30s at 58°C (primer annealing), 30 s at 72°C (signal acquisition); melting curve: 1 min at 95°C, 30s at 58°C, then continuous increase of temperature (0.11°C/s) to 95°C with continuous signal acquisition; Cool down: 10 s at 40°C. Melting curves showed a single peak with *B2M* primers rarely forming primer dimers in the negative controls. Telomere primers always form primer dimers due to the repetitive nature of their sequence. Evidence for primer dimer formation can be seen as melting peaks at slightly higher melting temperatures than the telomere qPCR product and also as amplification curves at very late cycles (average Cq for telomere negative controls: 38.1 (cattle) and 31.3 (sheep) compared to average Cq values of samples: 14.42 (SD = 0.76, cattle) and 13.52 (SD = 0.51, sheep)).

The software package LinRegPCR [23] was used to correct amplification curves for an estimated fluorescence baseline. The software also calculated well-specific amplification efficiencies. We used the mean efficiency across all wells on a plate, having excluded the upper and lower 5^th^ percentiles, as our reaction efficiency for each amplicon group [23]. The mean qPCR efficiencies across plates calculated with LinRegPCR ranged between 93.1%-94.2% (cattle) and 93.5%-94.0% (sheep) for the *B2M* reaction, and 93.6%- 94.4% (cattle) and 92.5%-95.5% (sheep) for the telomere reaction. We set a constant fluorescence threshold within the window of linearity across all plates for the calculation of Cq values. The threshold was for *B2M* 0.221 in cattle and 0.1 in sheep and for the telomere amplification 0.256 and 0.1 in cattle and sheep, respectively.

We calculated mean qPCR efficiencies separately for both amplicon groups (*B2M* and telomere reaction) for each qPCR plate using LinRegPCR. Samples were excluded from final analysis if at least one of their triplicate amplifications had a qPCR efficiency that was 5% higher or lower than the mean efficiency for the respective amplicon. Also, samples were excluded if their Cq values had a coefficient of variation (CV) > 5% across triplicates. Elimination of samples that failed quality control for qPCR efficiency or Cq values ensured high intra-plate repeatabilities and efficiencies, although less than 1% of our samples were excluded based on these criteria (see Table 1).

RTL was calculated using following formula described by Pfaffl [24]:

$$RTL=\frac{E_{TEL}^{Cq_{TEL\left(Calibrator\right)}-Cq_{TEL\left(Sample\right)}}}{E_{B2M}^{Cq_{B2M\left(Calibrator\right)}-Cq_{B2M\left(Sample\right)}}}$$

where E_TEL_ and E_*B2M*_ are the reaction efficiencies for the plate for the respective amplicon group calculated by LinRegPCR; Cq_TEL[Calibrator]_ and Cq_*B2M*[Calibrator]_ are the mean Cq values across triplicates for the telomere and *B2M* reactions, respectively, for the plate’s calibrator sample; and Cq_TEL[Sample]_ and Cq_*B2M*[Sample]_ are the mean Cq values across triplicates for telomere and *B2M* reactions, respectively, for the focal cattle or sheep sample.

An aim of our study was to test whether the use of a MS calibrator could control for differences in RTL amongst extraction methods. Therefore, in our initial cattle experiment we calculated RTL with the equation above but using three different calibrators: (1) a MS calibrator, (2) a calibrator extracted with a single method across all plates, arbitrarily choosing PG (termed "PG calibrator"), and (3) a constant Cq value across all plates (“no calibrator”). We chose constants of 26 for the reference gene and 14 for telomeres, as these were the average sample Cqs for these amplicons in our cattle experiment. The use of a constant Cq in the above equations allowed us to examine how well the use of a plate-specific calibrator (either MS or PG calibrators) accounted for plate-to-plate variation in RTL measures, whilst keeping RTL values on a similar scale as the RTLs calculated with MS and PG calibrators. In the subsequent sheep experiment, we only compared the MS calibrator with the no calibrator calculations (25.99 for reference gene and 13.71 for telomeres). We also examined variation in the raw Cq values for the telomere and *B2M* reactions. It is important to note that higher Cq values represent lower concentrations of telomere or reference gene and vice-versa in our RTL calculations.

### Statistical Analysis

Each sample was run on four identical qPCR plates per DNA extraction method. We calculated the Pearson’s correlation coefficient for the individual RTL measurements between all possible plate combinations. We took the average RTL for a sample across the four plates within each extraction method and calculated the Pearson’s correlation coefficient among methods. We calculated the CV–i.e. the standard deviation divided by the mean—across replicates of each sample both across all plates and within plate using the same extraction method. Pooled CVs across samples were calculated as the geometric mean CV.

Linear mixed models were used to estimate the repeatability of RTL measurements and Cq values for a given sample, the degree of plate to plate variation, and the effect of DNA extraction method on mean RTL. The model of analysis included the random effects of sample, sample-by-extraction method interaction and plate, and the fixed effect of DNA extraction method. Variance components for the random effects were estimated using restricted maximum likelihood. The sum all variance components constituted the total phenotypic variance. The repeatability of sample RTL across plates and methods was calculated as the ratio of the sample variance to the total phenotypic variance. The ratio of the sample-by-extraction method interaction to total phenotypic variance provided an estimate of the proportion of variance attributable to differences in RTL among extraction methods within a sample, whereas the ratio of the plate effect to total phenotypic variance expressed the proportion of variance attributable to differences in the mean RTL among plates. We tested the significance of any differences in mean RTL associated with DNA extraction method by comparing models with and without extraction method as a fixed effect using a likelihood ratio test. We ran separate models for RTL calculated using MS calibrators (both species), PG calibrators (cattle only) and no calibrator (both species). We made the same comparisons for the reference gene and telomere Cq values in both species. All statistical analyses were performed in R Studio with R 3.1.2 [25] with mixed-effects models being implemented using the ‘lme4’ library.

## Results

### DNA yield and integrity with different DNA extraction methods

A total of 56 of our PG and SC cattle samples, 51 of our SP cattle samples, and 36 of our sheep samples passed all quality controls for all DNA extraction methods and were used for RTL measurement (resulting in RTL measurements for a total of 235 DNA samples; Table 1). DNA yield was method dependent. The non-silica membrane-based PG extraction kit yielded the highest DNA concentrations (cattle: mean = 341 ± 6 ng/μl; sheep: mean = 282.6 ±2 ng/μl) and highest total yields of DNA (cattle: mean = 76 ± 2 μg; sheep: mean = 74 ±1 μg). The SC method produced substantially lower yields (cattle: mean concentration = 120 ± 2 ng/μl, mean total yield = 12 ± 0.2 μg; sheep: mean concentration = 68 ±1 ng/μl, mean total yield = 15 ±0.2 μg) and the SP method lower still (cattle: mean concentration = 38 ±0.6 ng/μl; mean total yield = 3 ±0.05 μg). However, initial whole blood volumes of cattle varied between DNA extraction methods (PG: 3 ml, SC: 600 μl, SP: 300 μl), whereas the same volumes of sheep buffy coat were used in all cases.

We also noticed that DNA integrity gels varied in appearance across extraction methods (Fig 1B). PG extracts showed the cleanest bands with no signs of smears and thus no signs of DNA disintegration. Based on our numeric integrity gel score (Fig 1A) all PG samples for both species scored a 1 (best score) while all spin column samples for sheep and 2 out of 69 samples for cattle scored a 2. Of the SP samples the majority of samples (83.9%) passed with a gel score of 2. A total of 11 SC or SP extracts from both species failed quality control based on their integrity gel score (Table 1).

### Repeatability of telomere length measurements & effects of DNA extraction method

**Fig 2:**
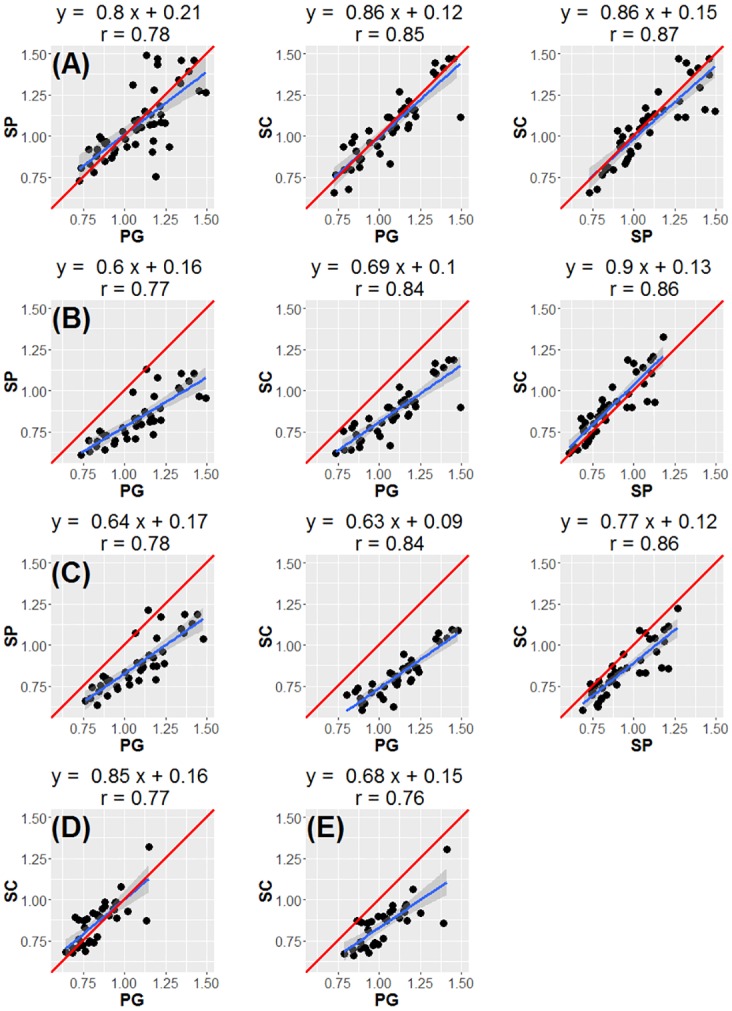
Correlations between methods. Correlations between RTL measurements from different DNA extraction methods (PG: Gentra Puregene kit; SC: DNeasy spin columns; SP: DNeasy 96 well plate): Cattle, method-specific calibrator (A); Cattle, Puregene calibrator (B); Cattle, no calibrator (C); Sheep, method-specific calibrator (D); Sheep, no calibrator (E). Regression lines and their 95% confidence interval are shown in blue and grey, respectively, with red lines reflecting a hypothetically perfect correspondence (slope of one, intercept of zero).

We found relatively high correlation coefficients and low CVs across plates for RTL measurements of the same sample in both species. All correlation estimates both within DNA extraction method (across plates) and between methods for the two species are summarized in S2 File. Correlations among RTL measurements from the same sample, calculated using a MS calibrator, among plates ranged from 0.87 to 0.96 for cattle, and 0.83 to 0.93 for sheep (S2 File). Correlations between average RTL measurements derived from different extraction methods and using different calibrators are summarised in Fig 2. Using a MS calibrator, correlations between the PG and SC methods were 0.85 for cattle and 0.77 for sheep, whilst in cattle the correlation between PG and SP was 0.78 and between SC and SP 0.87 (Fig 2). The correlation coefficients were comparable when a PG calibrator or no calibrator was used for RTL calculation (Fig 2). However, when fitting regression lines among samples extracted using different methods, application of the MS calibrator clearly produces regression slopes much closer to one with intercepts close to the origin (Fig 2). The average CV across all plates was 8.2% in cattle (12 plates, 3 methods), and 8.1% in sheep (8 plates, 2 methods). Within extraction method, CVs across plates were 9.2% and 8.2% for PG, 5.1% and 4.5% for SC, for cattle and sheep, respectively, and 5.2% for the SP in cattle only.

**Fig 3:**
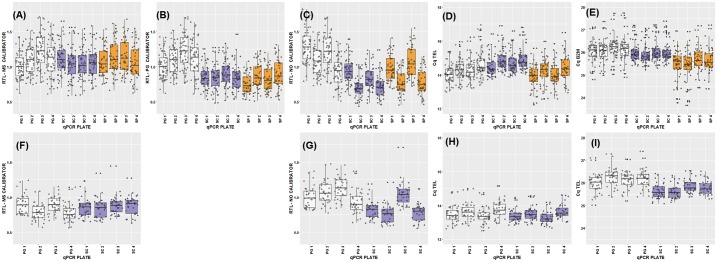
Raw RTL and Cq values. RTL or Cq values by DNA extraction method and qPCR plate for cattle (A-E) and sheep (F-I). RTL calculated with method specific (MS) calibrator (A + F), Puregene (PG) calibrator (B), no calibrator (C+G). Cq values for telomere reaction (D+H) and control gene *B2M* (E+I). Colours represent DNA extraction methods. White: Gentra Puregene, blue: DNeasy spin columns, orange: DNeasy 96 well plate.

In both cattle and sheep, we found significantly (P<0.05) higher mean RTL in samples extracted using the non-membrane-based method (PG) compared to those extracted with the silica membrane-based methods (SC and SP), when using either the PG calibrator or no calibrator in calculations (Fig 3, Table 2). This reflects genuine underlying differences in the average TL among DNA extracted from the same sample by different methods, as has been reported elsewhere [10,11]. These differences are underpinned by either or both lower telomeric Cq and higher reference gene Cq values in the PG extracted samples compared to the other methods (Fig 3D, 3E, 3H & 3I). In both species, there was notable variation in the telomeric Cq values across plates run on the same day, with the first plate having lower values than the second (Fig 3D & 3H). As would be expected, application of a plate-specific calibrator (either PG or MS calibrators) removed the within-day variation in RTL and substantially reduced among-plate variation (Table 2; Fig 3). Importantly, the differences in mean RTL among extraction methods became non-significant and sample repeatabilities were increased when a MS calibrator was used to calculate RTL (Fig 3, Table 2). This shows that using a MS calibrator to calculate RTL can account for observed effects of DNA extraction method on the underlying Cq values (Table 2; Fig 3).

**Table 2:** Variance component and parameter estimates.

	σ^2^_Total_	σ^2^_Sample_	σ^2^_SamplexMethod_	σ^2^_Plate_	σ^2^_Residual_	r^2^_Sample_	r^2^_SamplexMethod_	r^2^_Plate_	Χ^2^ Method	P Method	Effect PG vs. SC ± SE	Effect PG vs. SP ± SE
**Cattle**												
**RTL calculated with MS calibrator**	0.053	0.038	0.007	0.003	0.005	0.717	0.130	0.064	0.710	0.701	-0.035 ± 0.045	-0.016 ± 0.045
**RTL calculated with PG calibrator**	0.041	0.028	0.006	0.004	0.004	0.671	0.137	0.098	20.434	<0.001	-0.244 ± 0.048	-0.283 ± 0.048
**RTL calculated with no calibrator**	0.057	0.028	0.006	0.020	0.004	0.482	0.103	0.354	9.537	0.008	-0.330 ± 0.102	-0.237 ± 0.102
**Cq for *B2M* (reference gene)**	0.293	0.126	0.155	0.004	0.008	0.430	0.528	0.015	31.069	<0.001	-0.138 ± 0.089	-0.638 ± 0.091
**Cq for telomere amplification**	0.507	0.296	0.140	0.054	0.017	0.585	0.276	0.106	11.407	0.003	0.409 ± 0.179	-0.271 ± 0.180
**Sheep**												
**RTL calculated with MS calibrator**	0.020	0.012	0.003	0.002	0.002	0.615	0.162	0.113	0.620	0.431	0.027 ± 0.036	/
**RTL calculated with no calibrator**	0.034	0.014	0.004	0.012	0.003	0.430	0.113	0.359	4.815	0.028	-0.179 ± 0.079	/
**Cq for *B2M* (reference gene)**	0.150	0.051	0.083	0.013	0.003	0.338	0.555	0.084	11.185	0.001	-0.414 ± 0.105	/
**Cq for telomere amplification**	0.238	0.108	0.099	0.026	0.006	0.452	0.417	0.108	0.498	0.481	-0.091 ± 0.136	/

MS calibrator: Method specific calibrator, PG calibrator: Puregene extracted calibrator

## Discussion

In the present study, we addressed the effect of DNA extraction method on RTL measurements by comparing two silica membrane-based kits (SC and SP) with a kit that uses a non-membrane-based salting out method (PG). As expected [26], we found that the salting out method produced higher DNA yields and that silica membrane-based methods were associated with some observable loss of DNA integrity (Fig 1). A number of studies using human blood samples report significant differences in mean RTL depending on the DNA extraction method used [10–14]. We found that silica membrane-based DNA extraction methods produced shorter RTL measurements on average than the salting out method in both cattle and sheep. This is consistent with two previous studies in humans, which argued that silica membrane based DNA extraction methods reduce average RTL [10,11]. However, the physical and biochemical causes of these observed extraction method effects on RTL measurements are currently unknown, and determining these causes is an important next step for research in this area.

We found that the rank order of RTL measurements among samples is largely unaffected by DNA extraction methods. Across extraction methods, our RTL measures showed reasonably high repeatabilities and inter-plate correlations and low inter-plate CVs that were close to those reported in the qPCR telomere literature [7,14,16]. The aforementioned studies on human samples do not offer clear insight into how extraction methods affect the rank order of RTL measurements. One study reported relatively high correlations among samples extracted by QIAmp spin columns (QIAGEN) versus a magnetic bead extraction (Spearman’s ρ = 0.71) [11]; another study found a moderate correlation between a magnetic bead and a salting out extraction (Pearson’s r = 0.54)[14]. A third study found very low and not statistically significant correlations (r < 0.21) [12], and two of the studies did not present among sample correlations [10,13]. The absence of a strong correlation among RTL measurements based on different DNA extraction methods is a profoundly alarming result for research on telomere dynamics. If rank order of RTL is generally altered by underlying aspects of sample preservation, then associations among RTL and environmental, genetic and health measures within studies could themselves depend on the extraction method used. However, the one study reporting low correlations among RTLs based on different extraction method used DNA samples that would have failed our QC criteria [12] and it seems likely that the low correlations may be the result of variation in the level of DNA impurities that might have acted as qPCR inhibitors. Our results show that, as long as rigorous QC criteria are applied throughout telomere measurement protocols, the rank order of samples is very largely preserved regardless of the DNA extraction method used, despite the distribution of RTL estimates changing (Fig 2). Failure to carefully monitor and control the integrity and purity of DNA is likely to result in increased sampling error which will reduce the repeatability of results both within and among studies of telomere dynamics.

Importantly, our results show that it is possible to account for differences in mean RTL associated with DNA extraction method using a DNA extraction method-specific calibrator. Our reading of the literature suggests it is unusual for qPCR-based telomere studies in both epidemiology and ecology to provide much information about the source or preparation of the calibrator sample used. The five previous studies of DNA extraction method effects on RTL discussed above presumably used a calibrator sample extracted using only one extraction method, although most of them fail to explicitly state what kind of calibrator was used [10–13] and how it was extracted [10–14]. This is entirely reasonable given the aim was to test for differences in the telomere to control gene ratios associated with DNA extraction method. In this study, we have demonstrated a relatively simple approach that could account for DNA extraction method effects on RTL that could potentially allow researchers to perform qPCR based telomere studies combining samples extracted in different ways. By extracting large quantities of DNA from a single large sample of blood by different methodologies and running these on appropriate plates, we were able to apply an extraction method-specific calibrator in our calculations of RTL. This accounted for the extraction method effects on mean RTL which were observed in our two data sets when the standard calibration approach was used. More generally, our data suggest that within qPCR-based studies of TL, calibrator samples could be used for more than just accounting for plate to plate variation. As long as DNA integrity and purity is carefully controlled, calibrator samples derived from the same original sample but collected, stored or extracted in different ways could conceivably be used to control for systematic effects of variation in sample preparation on RTL.

It is obviously preferable to use a completely consistent approach and extract DNA using the same method within a study. However, a major challenge in the study of telomere dynamics is to generate sufficiently detailed longitudinal data to determine whether variation in TL observed later in life is the result of differences set in early life or differences in attrition rates across life [27]. Addressing this challenge in long-lived animals will inevitably require the use of long-term longitudinal archived samples, in which samples may have been stored or DNA extracted in different ways over time. Our calibrator-based approach could allow such valuable longitudinal samples to be compared within a single study, but it would need to be carefully validated each time it was applied. We would advocate applying similarly stringent quality control on DNA integrity and purity as here, even though this may reduce available sample size. Before applying a method-specific calibrator approach to archived samples prepared in different ways, it would also be crucial to run a similar experiment to establish the repeatability of RTL measures among samples that have been experimentally exposed to the relevant differences in sample collection, storage or DNA extraction.

## Conclusion

This study adds to the emerging literature showing that DNA extraction methods may affect the mean RTL measurement produced by qPCR techniques. We present the first evidence for such effects in non-human vertebrates, documenting similar results in two ruminant species of considerable economic and agricultural importance in which TL variation has recently been examined with some exciting initial results [28–30]. We also show that RTL measurements derived from different DNA extraction methods are highly correlated when rigorous DNA quality control is applied. Our results also suggest that the application of method-specific calibration in qPCR studies of RTL could allow researchers to effectively use valuable historical archives of samples that have been prepared or extracted in different ways, accounting for effects of methodological variation on mean RTL.

## Supporting Information

S1 File Modified DNA extraction protocols. (DOCX)

S2 File Correlations within and between DNA extraction methods. (DOCX)
