# Supplementary material for: Method Specific Calibration Corrects for DNA Extraction Method Effects on Relative Telomere Length Measurements by Quantitative PCR
Source: PLoS One. 2016 Oct 10;11(10):e0164046. doi: 10.1371/journal.pone.0164046 (PMC5056729; doi:10.1371/journal.pone.0164046)
Supplement: S1 File — (DOCX) [file pone.0164046.s001.docx]

# Supplementary File 1

# Protocols for the extraction of DNA from bovine whole blood and ovine buffy coat

Last Update: 01.06.2014

## DNA Extraction from bovine whole blood

### Salting out using Gentra puregene kit (QIAGEN)

1. **Thaw** whole blood samples slowly at **4°C** (takes for 1-5 ml approximately 2-3 hours).

(N.B. The manufacturer’s manual recommends thawing quickly in the water bath at 37 °C, but slow thawing might help if DNA yields tend to be low.)

1. Dispense **9 ml RBC Lysis Solution** into a 15 ml centrifuge tube.
2. Add **3 ml whole blood** and mix by **inverting 10 times**.
3. Incubate **5 min at room temperature** (15-25°C). **Invert** at least once during incubation.
4. Centrifuge for **2 min at 2000 x g** to pellet the white blood cells.
5. **CAREFULLY pipette off supernatant** (10 ml pipette, then 1 ml pipette) leaving approximately **200 μl of the residual liquid** and the white blood cell pellet.
6. Vortex the tube vigorously to resuspend the pellet in the residual liquid.
   *Vortexing greatly facilitates cell lysis in the next step. The pellet should be completely dispersed after vortexing.*
7. Add **3 ml Cell Lysis Solution** and **pipet up** and down to lyse cells. **Vortex vigorously for 10 seconds.**

*Samples are very stable at that stage (up to 2 years at room temperature) and can be left in the fridge overnight.*

1. Set **water bath to 37°C**.
2. Add **15 μl RNAse A Solution** (QIAGEN) and mix by **inverting 25 times**. **Incubate for 15 min at 37°C**.
3. Incubate for **3 min on ice** to quickly cool down the samples.
4. Set **water bath to 65°C**.
5. Add **1 ml of Protein Precipitation Solution**, and **vortex vigorously for 20s** at high speed.
6. **Centrifuge for 5 min at 2000 x g.**

*The precipitated protein should form a tight, dark brown pellet. If not, pipet up and down, incubate sample on ice for 5 min and repeat centrifugation.*

1. Pipet **3 ml isopropanol into a clean 15 ml tube** and **add the supernatant** from the previous step by pipetting (10 ml pipette).
   *Be sure the protein pellet is not dislodged.*
2. **Mix by inverting gently 50 times** until the DNA is visible as threads or a clump.
3. **Transfer DNA and 1 ml of liquid to a 1.5 ml microcentrifuge tube** (use 1 ml pipette with broad tips)

(N.B. This is the main alternation of the original protocol where the DNA pellet stays in 15 ml falcon tubes during all washing end elution steps. We found that it is less likely to lose DNA if the pellet is transferred to a microcentrifuge tube and if the microcentrifuge protocol provided by the manufacturer is followed until DNA elution. )

1. Centrifuge **1 min at 13 000 x g**.
2. **Pipette off isopropanol** (centrifuge again and pipette off residual).
3. Wash with **500 μl 70% Ethanol**.
4. Centrifuge **1 min at 13 000 x g**, **pipette off Ethanol**, centrifuge again and pipette off residual).
5. **Dry until pellet gets a bit translucent** (~2-8 min).
6. Add **DNA hydration solution** to dissolve gDNA (50 – 400 μl depending on pellet size).
7. Incubate at **65°C for 1** hour to dissolve gDNA.
8. Incubate at **room temperature overnight** on **rotator**.
9. Measure DNA yield and quality (260/280, 260/230) on the next day (NanoDrop).
10. Samples are stable at 4°C indefinitely but for long term storage put at – 20°C.

(Protocol can be started with step 9 for samples that have been stored overnight in Cell Lysis Solution. During the following steps the next lot of samples can thaw in the fridge. They can be prepared until step 8 during step 24.)

### Silica membrane based extractions (DNeasy Blood & Tissue Kit by QIAGEN)

#### I.II.I. Spin column protocol

**Step 1.-12. are prepared in duplicates to increase DNA yield. Therefore, do step 14. with the first duplicate, place spin column in a new 2 ml microcentrifuge tube, add second duplicate and centrifuge again for 1 min at 8800 rpm. Discard flow through and collection tube and continue with step 15.**

1. **Thaw** whole blood samples slowly at **4°C** (takes for 1-5 ml approximately 2-3 hours).

(N.B. The manufacturer’s manual recommends thawing quickly in the water bath at
37 °C, but slow thawing might help if DNA yields tend to be low.)

1. Dispense **900 μl RBC Lysis Solution** into a 2 ml microcentrifuge tube.

(N.B. We added this additional step similar to the beginning of the Puregene extraction protocol to the beginning of the DNeasy extractions where we lysed and removed red blood cells, keeping only the leukocyte pellet for the subsequent DNA extraction. Before, a low OD 260/230 ratio implied salt contamination for which EDTA could have been the reason. By removing a majority of protein and salts before samples were applied to silica membranes, both NanoDrop ratios for purity measurement improved notably indicating an improvement in DNA purity due to the changes of the protocol. We added steps 1.-7. to the manufacturer’s protocol.)

1. Add **300 μl whole blood** and mix by **inverting 10 times**.
2. Incubate **5 min at room temperature** (15-25°C). **Invert** at least once during incubation.
3. Centrifuge for **30 s at 14 000 x g** to pellet the white blood cells.
4. **CAREFULLY pipette off supernatant** (1 ml pipette) leaving approximately **10 μl of the residual liquid** and the white blood cell pellet.
   Vortex the tube vigorously to resuspend the pellet in the residual liquid.

*Vortexing greatly facilitates cell lysis in the following steps. The pellet should be completely dispersed after vortexing.*

1. Dispense cell pellet in **90 μl PBS** by pipetting up and down and vortexing.
2. Add **30 μl Proteinase K.**

**(N.B.** We increased the volume of Proteinase K from 20 to 30 μl to improve purity of the extracts further.)

1. Add **4 μl RNase A** (100 mg/ml) and incubate for 2 min at room temperature.
2. Add **300 μl** Buffer AL (without added ethanol). Mix thoroughly by vortexing, and incubate at **56°C for 10 min**.

*Ensure that ethanol has not been added to Buffer AL (see “Buffer AL”, page 18). Buffer AL can be purchased separately (see page 56 of the manufacturer’s hand book for ordering information).*

*It is essential that the sample and Buffer AL are mixed immediately and thoroughly by vortexing or pipetting to yield a homogeneous solution*

(N.B. Instead of 200 μl we used 300 μl buffer AL to promote cell Lysis. For the same reason we repeated the following incubation step at 56 °C after vortexing the samples thoroughly in step 38.)

1. Vortex samples for **10 s** and incubate for **10 more minutes at 56°C**.
2. Add **200 μl ethanol (96–100%)** to the sample, and mix thoroughly by vortexing.

*It is important that the sample and the ethanol are mixed thoroughly to yield a homogeneous solution*

1. Place **AE buffer** in the water bath at **56°C.**
2. Pipet the mixture from step 12 into the **DNeasy Mini spin column placed in a 2 ml collection tube** (provided). Centrifuge at **8800 rpm for 1 min**. Discard flow-through and collection tube.^[[1]](#footnote-1)^ (If duplicates were prepared, repeat this step with the second duplicate on the same spin column.)
3. Place the DNeasy Mini spin column in a new 2 ml microcentrifuge tube (not provided), add **300 μl Buffer AW1**, and wash the inside of the DNAesy Mini spin column thoroughly by **inverting each tube 5 times and then roll them in your fingers 5 times**.
   Centrifuge for **1 min at 8800 rpm**. Discard flow-through and collection tube. ^1^

(N.B. Both washing steps with buffer AW1 and AW2 were performed with 300 μl buffer (instead of 500 μl), but they were repeated once each. Rolling and inverting the tubes containing the washing buffer ensured that the inside of the tubes including the lids were reached and cleaned by the buffers.)

1. **Repeat step 15**.
2. Place the DNeasy Mini spin column in a new 2 ml collection tube (provided), add **300 μl Buffer AW2**, and wash the inside of the DNAesy Mini spin column thoroughly by **inverting each tube 5 times and then roll them in your fingers 5 times**.
   Centrifuge for **3 min at 14,000 rpm (20,000 x g)** to dry the DNeasy membrane. Discard flow-through and collection tube.

   *It is important to dry the membrane of the DNeasy Mini spin column, since residual ethanol may interfere with subsequent reactions. This centrifugation step ensures that no residual ethanol will be carried over during the following elution.*
3. **Repeat step 17**.
4. *Following the centrifugation step, remove the DNeasy Mini spin column carefully so that the column does not come into contact with the flow-through, since this will result in carryover of ethanol. If carryover of ethanol occurs, empty the collection tube, then reuse it in another centrifugation for 1 min at 20,000 x g (14,000 rpm).*
5. Place the DNeasy Mini spin column in a clean 1.5 ml microcentrifuge tube (not provided), and pipet **100 μl Buffer AE (warm; see step 13) directly onto the DNeasy membrane**.

(N.B. To increase the elution efficiency the elution buffer AE was warmed to 56°C before 100 μl were added onto the silica membranes. Samples were then incubated for 10 min at 56°C in a heat block before final centrifugation for elution.)

1. Incubate at **56°C for 10 min** (use heat block to prevent condensation water in the lids), and then centrifuge for **1 min at 8800 rpm** to elute.
2. If samples were prepared in duplicates, do two elution steps: Pipet **50** **μl warm Buffer AE** directly onto the DNeasy membrane, incubate at **56°C for 5 min** (use heat block) and centrifuge for **1 min at 8800 rpm**.

*If you want to use more than 200* μl buffer AE in total to elute the DNA of a single sample, use a new microcentrifuge tube for the second elution step. Otherwise, the silica membrane will touch the flow through.

#### I.II.II 96 Well Plate

N.B. Red blood cells are lysed and white blood cells are spun down and collected for DNA extraction prior to the beginning of the manufacturer’s protocol. Because preparation of samples in this way was not successful in deep 96 well plates, samples are prepared in micro-centrifuge tubes until they are transferred onto silica membranes. From this step onwards the manufacturer’s protocol is followed with minor alternations:

1. **Thaw** whole blood samples slowly at **4°C** (takes for 1-5 ml approximately 2-3 hours; can be done overnight).
2. Dispense **900 μl RBC Lysis Solution** into a 1.5 ml micro-centrifuge tube (not provided by the kit).
3. Add **300 μl whole blood, seal plate** and mix by **inverting 10 times**.
4. Incubate **5 min at room temperature** (15-25°C). **Invert** at least once during incubation.
5. Centrifuge for **30 s at 14 000 x g** to pellet the white blood cells.
6. **CAREFULLY pipette off supernatant** (1 ml pipette) leaving approximately **10 μl of the residual liquid** and the white blood cell pellet.
   Vortex vigorously to re-suspend pellet in residual liquid.

*Vortexing greatly facilitates cell lysis in the following steps. The pellet should be completely dispersed after vortexing.*

1. **Re-suspend white blood cell pellet in 190 μl PBS** and **transfer.**
2. Add **20 μl proteinase K** to each sample and mix thoroughly.
3. Add **4 μl RNase A** (100 mg/ml) and incubate for **5 min at room temperature**.
4. Add **200 μl** Buffer AL (without added ethanol) to each sample and mix by pipetting up and down.
5. Vortex **vigorously for 15 s**.
6. Incubate at **56°C for 10 min**. Mix occasionally during incubation to disperse the sample, or place on a rocking platform.
7. Add **200 μl ethanol (96–100%)** to each sample.
8. Vortex **vigorously for 15 s**.
9. **Place two DNeasy 96 plates on top of S-Blocks** (provided by kit). Mark the DNeasy 96 plates for later sample identification
10. **Carefully transfer the lysis mixture (maximum 900 μl) of each sample from step 16 to each well of the DNeasy 96 plates**. Take care not to wet the rims of the wells to avoid aerosols during centrifugation. Do not transfer more than 900 μl per well.

Note:
Lowering pipet tips to the bottoms of the wells may cause sample overflow

and cross-contamination. Therefore, remove one set of caps at a time, and begin

drawing up the samples as soon as the pipet tips contact the liquid. Repeat until all the samples have been transferred to the DNeasy 96 plates.

1. **Seal each DNeasy 96 plate with an AirPore Tape Sheet** (provided by kit). **Centrifuge for 4 min at 6000 rpm**.

   AirPore Tape prevents cross-contamination between samples during centrifugation. After centrifugation, check that all of the lysate has passed through the membrane in each well of the DNeasy 96 plates. If lysate remains in any of the wells, centrifuge for a further 4 min.
2. Remove the tape. Carefully **add 500 μl Buffer AW1** to each sample.

**Note:**
Ensure that ethanol has been added to Buffer AW1 prior to use.

1. **Seal each DNeasy 96 plate with a new AirPore Tape Sheet** (provided by kit). **Centrifuge for 2 min at 6000 rpm (=5796 x g)**.
2. **Repeat steps 18-19.**
3. Remove the tape. Carefully **add 500 μl Buffer AW2** to each sample.

**Note:**
Ensure that ethanol has been added to Buffer AW2 prior to use.

1. Place **buffer AE** in the water bath at **56°C.**
2. **Centrifuge DNeasy Plates for 15 min at 6000 rpm.
   Do not seal the plate with AirPore Tape.**
   The heat generated during centrifugation ensures evaporation of residual ethanol in the sample (from Buffer AW2) that might otherwise inhibit downstream reactions.
3. Place each DNeasy 96 plate in the correct orientation on a **new rack of Elution Microtubes RS** (provided by kit).
4. To elute the DNA, add **80 μl Buffer AE** (warm; see step 24) to each sample, and seal the DNeasy 96 plates with new AirPore Tape Sheets (provided by kit).
   Incubate for 1 min at room temperature (15–25°C) (or better **for 10 min at 56 °C**). **Centrifuge for 4 min at 6000 rpm.**

   (200 μl Buffer AE is sufficient to elute up to 75% of the DNA from each well of the DNeasy 96 plate. Elution with volumes less than 200 μl significantly increases the final DNA concentration of the eluate but may reduce overall DNA yield. For samples containing less than 1 μg DNA, elution in 50 μl Buffer AE is recommended.)
5. **Recommended:** For maximum DNA yield, repeat step 27 with another 80 μl Buffer AE.

   (A second elution with Buffer AE will increase the total DNA yield by up to 25%. However due to the increased volume, the DNA concentration is reduced. If a higher DNA concentration is desired, the second elution step can be performed using the 200 μl eluate from the first elution. This will increase the yield by up to 15%. Use new caps (provided) to seal the Elution Microtubes RS for storage.)

## Extraction from ovine buffy coat using Gentra Puregene Kit (QIAGEN)

*Most alternations were done to allow working with Eppendorf tubes rather than with
15 ml falcon tubes. For being able to prepare samples in tubes that are so much smaller,
volumes of some reagents had to be adjusted. Also, centrifugation times were adapted
to micro- centrifuges.*

1. Thaw buffy coat quickly in 37^o^C water bath, store on ice. (change WB temp to 55^o^C)
2. Aliquot 100μl buffy coat into 2ml Eppendorf micro- centrifuge tube. Return remaining
   stock buffy to correct box in -20°C, having marked to indicate that it has been thawed.

   *Because of high DNA yields extractions were started with less buffy coat then
   recommended in the manufacturer’s manual (150-200 μl)*.
3. Add 600μl RBC lysis solution, pipette up and down to mix.

   *Red blood cells were lysed by adding 600 μl instead of 300 μl red blood cell lysis solution
   to ensure lysis of red blood cells.*
4. Incubate for 10 min at room temperature with mixing if there are lumps.
5. Centrifuge 3 min at 13000 x g.
6. Pipette off supernatant leaving ~ 150ul. Re-suspend pellet until no lumps are visible by tube flicking and vortexing. Avoid using a pipette, to prevent losing material in tip.
7. Add 1ml cell lysis solution, pipette and vortex to mix.
8. Incubate 1 hour at 55°C to dissolve all lumps. If still viscous and lumpy, add 1-2ul proteinase
   K to further digest.
9. Add 10ul RNase solution, mix by inverting. Incubate 37^o^C for 15min.
10. Chill on ice 3mins.
11. Add 400ul protein precipitation solution and vortex for 20 secs at high speed.
12. Centrifuge 3min at 13000g
13. Pipette supernatant into 2x 500ul isopropanol. Invert until strings of gDNA are visible.
    *The manufacturer’s protocol recommends using 3 ml of Isopropanol in a 15 ml falcon tube,
    but we found that 1 ml is sufficient to pellet the DNA and that working with Eppendorf tubes reduces the risk to lose the DNA pellet.*
14. Centrifuge 1 min 13000g. Use pipette to remove excess isopropanol.
15. Wash with 400ul 70% EtOH.
16. Centrifuge 1min 13000g, pipette off EtOH. Leave upturned on paper towel on bench to dry for 4-5 minutes, or until pellet is translucent.
17. Add DNA hydration solution to dissolve gDNA (100-300ul depending on pellet size), then incubate at 65°C 1hour to dissolve gDNA.
18. Leave overnight on rotator and measure on Nanodrop the next day. Samples are then stable 4^o^C indefinitely but store long-term at -20^o^C.

1. Flow-through contains Buffer AL or Buffer AW1 and is therefore not compatible with bleach. See page 8 of the DNeasy handbook for safety information. [↑](#footnote-ref-1)
