# Supplementary material for: Method Specific Calibration Corrects for DNA Extraction Method Effects on Relative Telomere Length Measurements by Quantitative PCR
Source: PLoS One. 2016 Oct 10;11(10):e0164046. doi: 10.1371/journal.pone.0164046 (PMC5056729; doi:10.1371/journal.pone.0164046)

# Supplementary File 2: Correlation Matrices

Correlation matrices within (across plate) and between DNA extraction methods

Explanation of axis labels:

| SP | Spin Plate extracts (qPCR plates 1-4) |
| --- | --- |
| PG | Puregene extracts (qPCR plates 1-4) |
| SC | Spin Column extracts (qPCR plates 1-4) |
| MS.cal | Method-specific calibrator |
| PG.cal | Puregene calibrator |
| No.cal | No calibrator |
| average | Average RTL values calculated across 4 plate repeats |

The colour bar underlying each plot explains the colour coding that was applied to visualise the strength and direction of individual correlations. No correlation would appear as white field, negative correlations as different shades of red, and positive correlations as different shades of blue. It can be seen very quickly that all correlations are positive and high.

N.B. Correlation coefficients do not exactly agree with figure 2, because only complete observations (without missing values) can be used for the calculation of correlation matrices. Therefore, for cattle they are based on 44 samples, whereas correlation coefficients shown in figure 2 are based on up to 56 samples. For sheep correlation matrices were calculated using 35 complete observations (out of 42).

## Cattle

Correlations among RTL measurements calculated using a method-specific calibrator


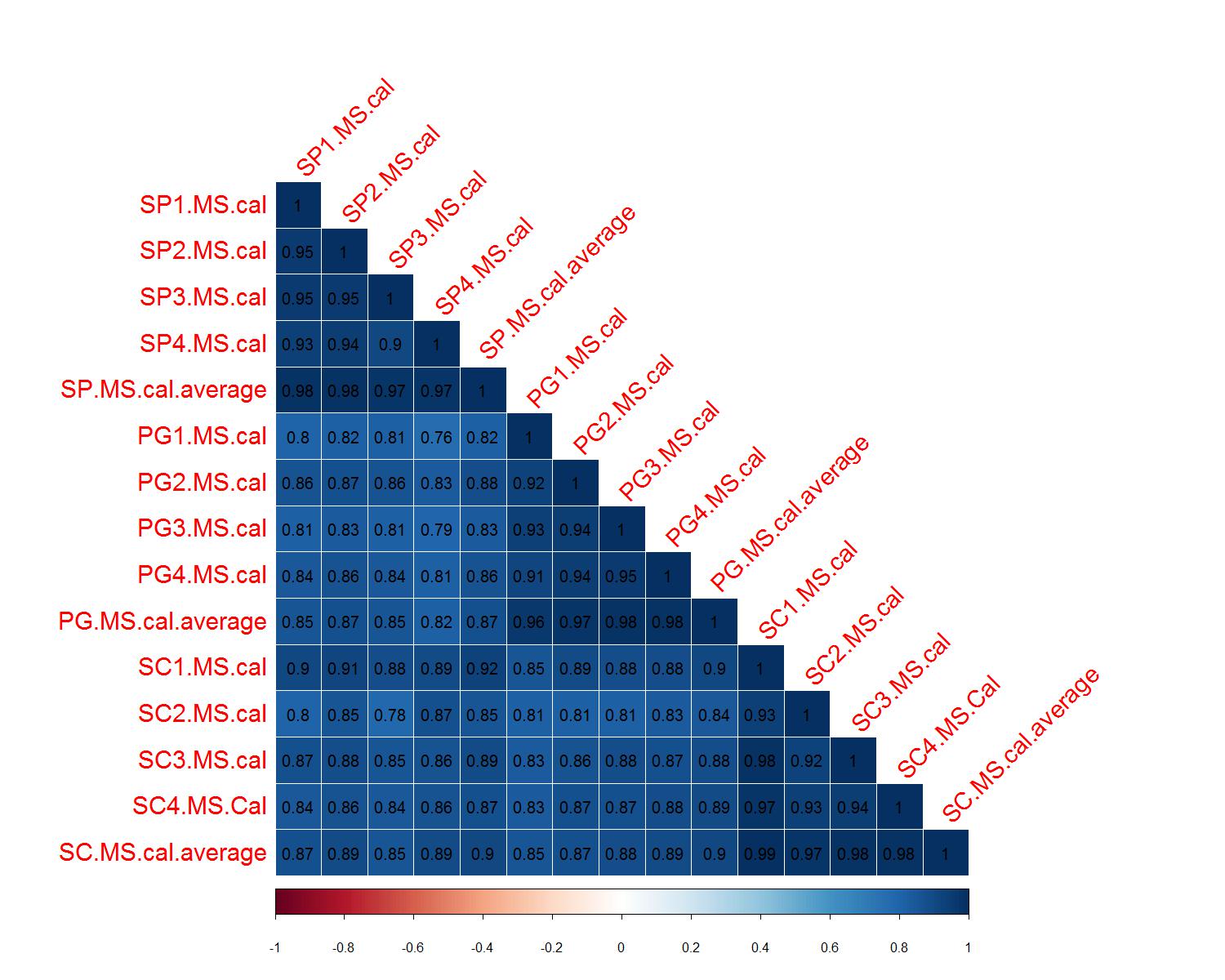


Correlations among RTL measurements calculated using a Puregene extracted calibrator
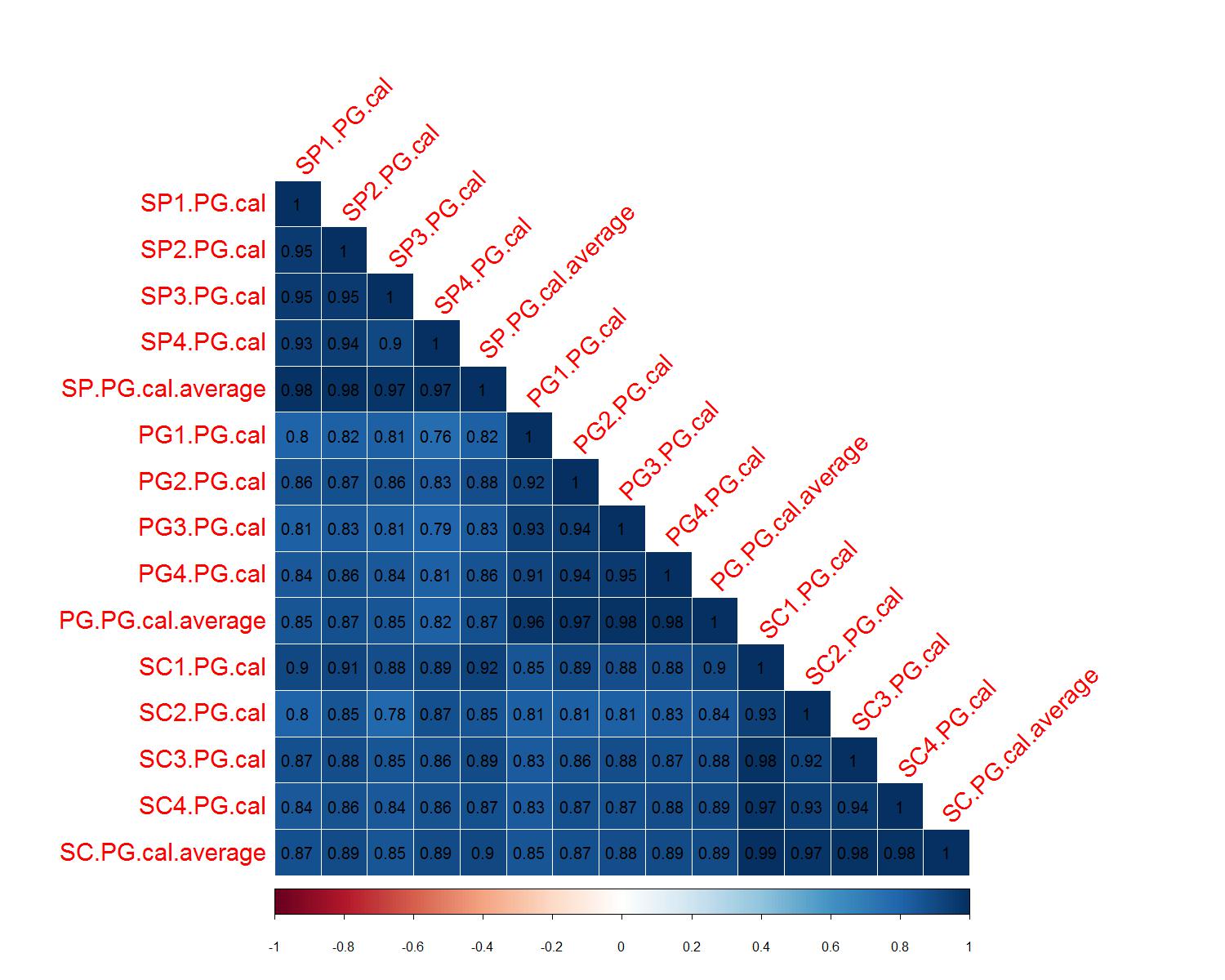


Correlations among RTL measurements derived without a calibrator


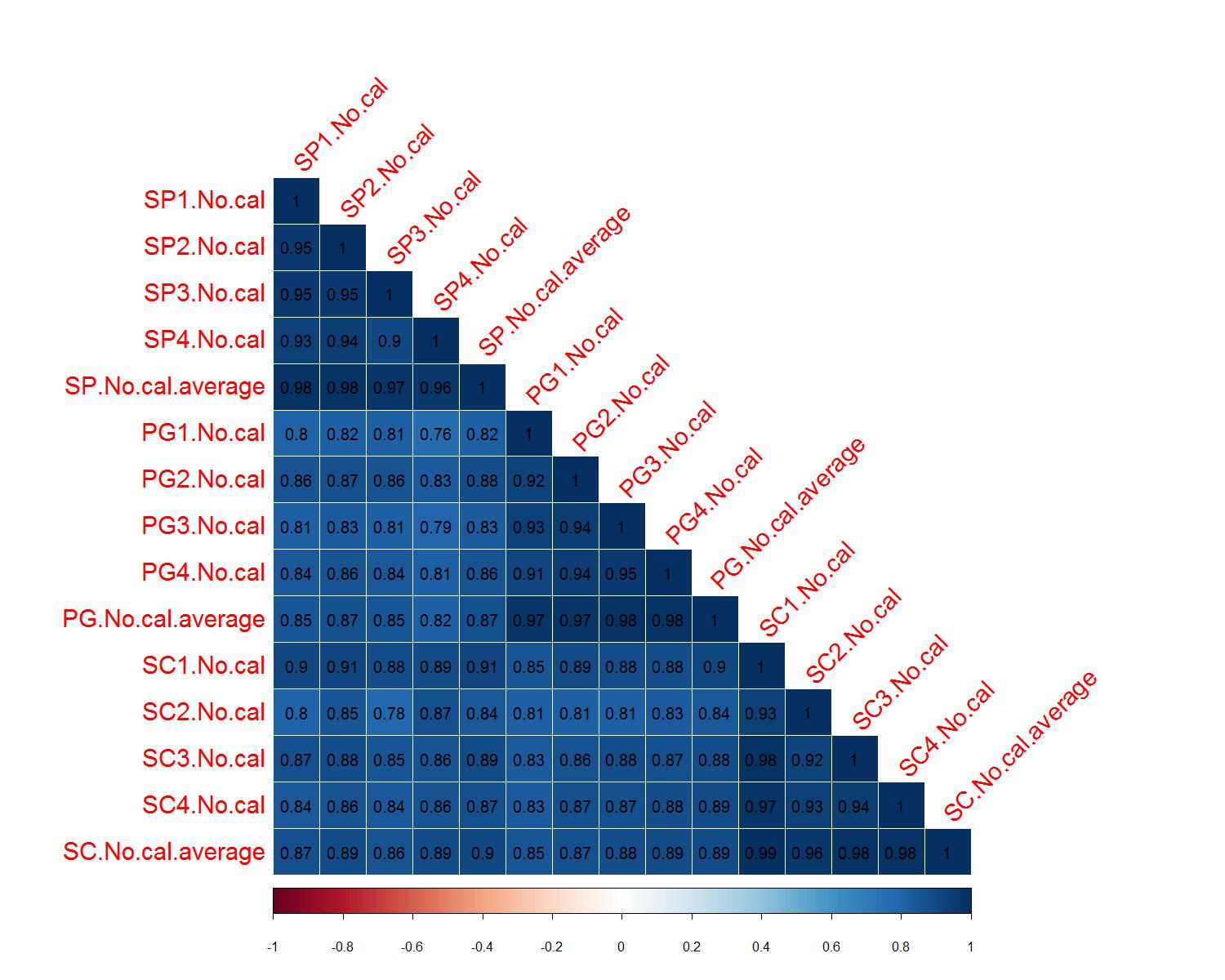


## Sheep

Correlations among RTL measurements calculated using a method-specific calibrator


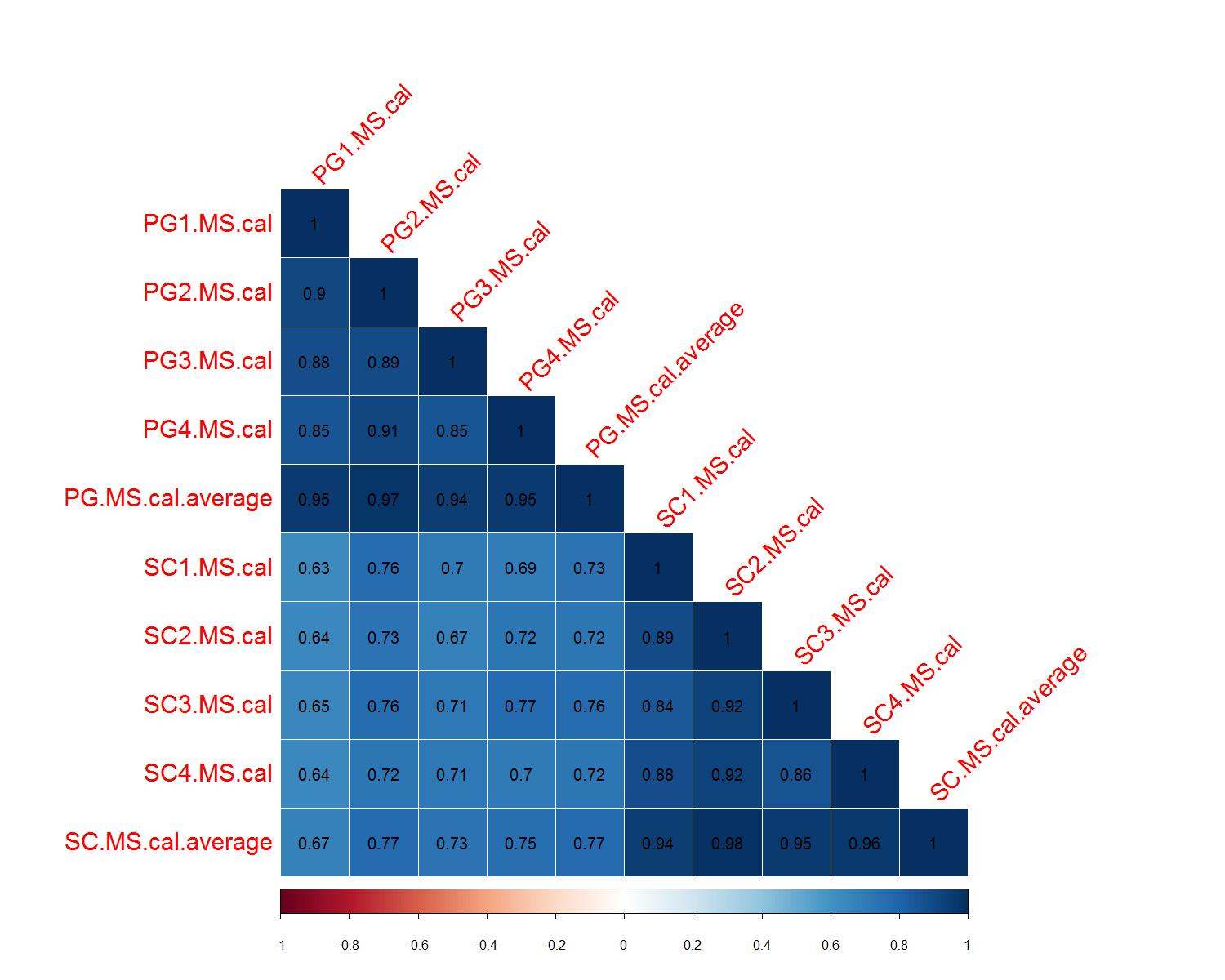


Correlations among RTL measurements derived without a calibrator


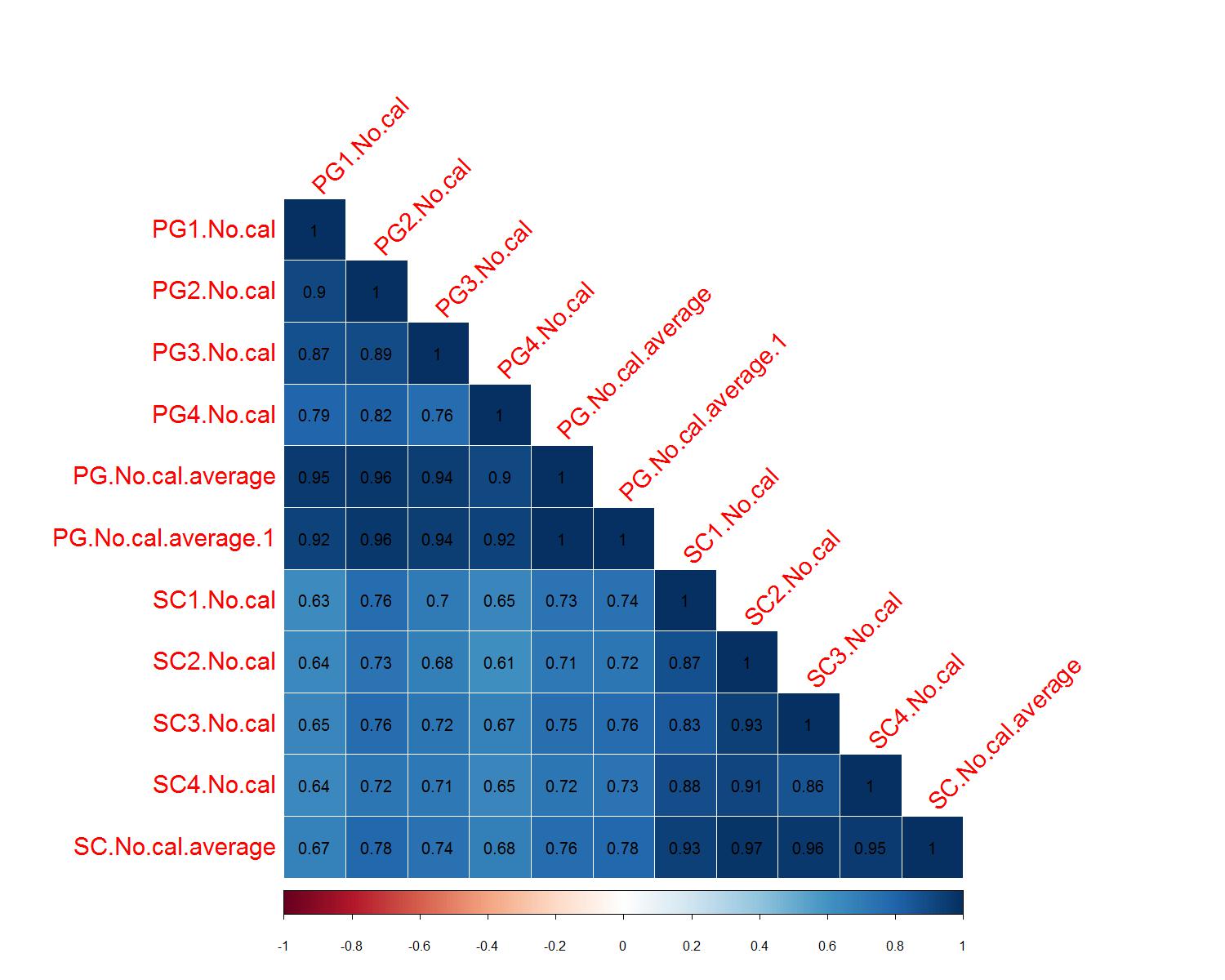

Supplement: S2 File — (DOCX) [file pone.0164046.s002.docx]
